# Detection of DNA fusion junctions for *BCR*-*ABL *translocations by Anchored ChromPET

**DOI:** 10.1186/gm191

**Published:** 2010-09-22

**Authors:** Yoshiyuki Shibata, Ankit Malhotra, Anindya Dutta

**Affiliations:** 1 Department of Biochemistry and Molecular Genetics, University of Virginia, School of Medicine, 1300 Jefferson Pk Ave, Charlottesville, VA 22908-0733, USA

## Abstract

Anchored ChromPET, a technique to capture and interrogate targeted sequences in the genome, has been developed to identify chromosomal aberrations and define breakpoints. Using this method, we could define the *BCR*-*ABL1 *translocation DNA breakpoint to a base-pair resolution in Philadelphia chromosome-positive samples. This DNA-based method is highly sensitive and can detect the fusion junction using samples from which it is hard to obtain RNA or cells where the RNA expression has been silenced.

## Background

Chromosomal translocations play a major role in several genetic diseases. Translocations between genes have the potential to constitutively express or repress genes and hence lead to different diseases. The Philadelphia chromosome (Ph) is a prime example of such a translocation, where a fusion gene is constitutively expressed and leads to a particular class of leukemia. There are other translocations that have been implicated in cancers and other genetic diseases, and more are being discovered every day. A method that can quickly and robustly characterize specific translocations and produce DNA-based disease-specific biomarkers will have both diagnostic and prognostic applications. A method that is not dependent on the growth of cells in culture will bring the power of cytogenetics to many more cancers.

The incidence of chronic myeloid leukemia (CML) is 1 to 2 per 100,000 and the disease constitutes 15 to 20% of adult leukemias. CML is characterized by the Ph, resulting from the t(9;22)(q34;q11) balanced reciprocal translocation. The translocation generates the BCR-ABL1 fusion protein with constitutive kinase activity and oncogenic activity. The breakpoints in the *ABL1 *gene lie in a 90-kb-long intron 1, upstream of the ABL1 tyrosine kinase domains encoded in exons 2 to 11. The breakpoints within *BCR *are mapped to a 5.8-kb area spanning exons 12 to 16, the major breakpoint cluster region (M-bcr), found in 90% of patients with CML and in 20 to 30% of patients with Ph-positive B-cell acute lymphoblastic leukemia (Ph+ B-ALL) [1-3].

Detection of Ph or *BCR*-*ABL1 *transcripts establishes a diagnosis of CML or Ph+ B-ALL. The majority of CML patients are in the chronic phase of the disease when they have their blood tested for diagnosis. Most patients in the chronic phase are treated for extended periods of time by inhibitors of BCR-ABL1 tyrosine kinase, such as imatinib mesylate [4-6]. These patients must be monitored continuously to follow their response to drugs and to ensure that the disease does not recur. Generally, a white blood cell count is performed as a routine laboratory examination. A chemical profile also gives important information. However, cytogenetics is still considered the gold standard for diagnosing CML and evaluating the response to therapy. There are two major forms of cytogenetic testing. Karyotyping requires condensation of chromosomes and thus cells undergoing mitosis. Therefore, karyotyping is usually done on bone marrow aspirates, with the cells being cultured for several days to increase their number and to ensure active cell cycling before arrest in metaphase. The *in vitro *cell culture step is essential for karyotyping. Another method of cytogenetic testing is fluorescent *in situ *hybridization (FISH), which can be applied to nondividing cells isolated from peripheral blood. FISH is able to detect *BCR*-*ABL1 *translocation directly with fluorescent-labeled DNA probes and allows the detection of the *BCR*-*ABL1 *fusion gene in some cytogenetically Ph-negative cases with microscopically invisible rearrangements of chromosomes 9 and 22 [7-10]. However, neither karyotyping nor interphase FISH yields a sensitive and convenient molecular biomarker that can be used for follow-up of patients during treatment.

Real-time reverse transcription PCR (RT-PCR) is the most sensitive technique available for the detection of *BCR*-*ABL1 *transcripts and is used to follow the progression of CML after initial diagnosis and treatment [11]. Although RT-PCR detects *BCR*-*ABL1 *transcripts from a small number of cells, the quality and efficiency of RNA extraction and/or reverse transcription affect the result. False negative cases may arise from degradation of the RNA following the harvesting of patient cells or from repression of the *BCR*-*ABL1 *transcript. In fact, an important question in the treatment of CML is whether a negative result in the RT-PCR test means that the patient is truly free of the disease and can be taken off imatinib treatment. Mattarucchi *et al*. [12] reported the persistence of leukemic DNA even with undetectable levels of chimeric transcript. Thus, a DNA-based marker of the translocation will facilitate patient management by confirming the absence of leukemic DNA. In addition, genetic heterogeneity is known among patients with CML and it is unclear whether the chromosomal translocation breakpoint influences disease progression because there has not been an easy method to sequence such breakpoints [13].

Here we introduce a method for detecting and monitoring the *BCR*-*ABL1 *translocation based on a screen for the DNA breakpoint. As demonstrated previously, paired-end tags (PET) technology is a powerful technique to identify unconventional fusion transcripts and structural variations in the genome [14-18]. However, a genome-wide approach to detect the *BCR*-*ABL1 *translocation for CML diagnosis is still too costly in both time and money. Anchored ChromPET combines three critical techniques: capture of a targeted region to selectively enrich the region of interest, chromosomal PET (chromPET) sequencing to interrogate the genomic locus, and bar-coding to multiplex multiple samples into a single ultra-high-throughput sequencing lane. Using the M-bcr as a model, we demonstrate the usefulness of this technique for obtaining the sequence of the *BCR*-*ABL1 *DNA translocation junction from multiple samples in a single lane of the Illumina genome analyzer II (GA-II). The high resolution of breakpoint identification, production of a patient-specific DNA biomarker, and the stability of DNA relative to RNA suggest that Anchored ChromPET will be useful for the detection and follow-up of diseases such as CML that are caused by specific chromosomal translocations.

## Materials and methods

### Reagents

Reagents used were APex Heat-Labile Alkaline Phosphatase (Epicentre, Madison, WI, USA; AP49010), Biotin-16-UTP (Roche, Indianapolis, IN, USA; 11388908910), DNAZol reagent (Invitrogen, Carlsbad, CA, USA; 10503-027), Dynabeads M-280 streptavidin (Invitorgen; 112-05D), End-It DNA End Repair Kit (Epicentre; ER0720), human Cot-1 DNA (Invitrogen; 15279-011), MAXIscript Kit (Ambion, Austin, TX, USA; AM1312), MinElute Reaction Cleanup Kit (Qiagen, Valencia, CA, USA; 28204), pCR4-TOPO-TA vector (Invitrogen; K4575-01), QIAquick Gel Extraction Kit (Qiagen; 28704), QIAquick PCR Purification Kit (Qiagen; 28104), QuickExtract FFPE DNA Extraction Kit (Epicentre; QEF81805), QuickExtract FFPE RNA Extraction Kit (Epicentre; QFR82805), Quick Ligation Kit (NEB, Ipswich, MA, USA; M2200S), SuperScript III Reverse Transcriptase (Invitrogen; 18080-093), TaKaRa Ex Taq DNA Polymerase (Takara, Otsu, Shiga, Japan; TAK RR001A), Taq DNA Polymerase (Roche; 11146165001), TRIzol (Invitrogen; 15596-026), and TURBO DNase (Ambion; AM2238).

### Cell lines

K562 cells (CCL-243) and KU812 cells (CRL-2099) were purchased from ATCC and cultured according to ATCC instructions.

### Patient samples

Genomic DNA from peripheral blood mononuclear cells were kindly provided by Dr Brian Druker (Oregon Health and Science University). Ph+ or Ph- patient samples were obtained with informed consent and under the approval of the Oregon Health and Science University Institutional Review Board. Mononuclear cells were isolated by separation on a Ficoll gradient (GE Healthcare, Piscataway, NJ, USA), followed by purification of genomic DNA using the Dneasy Blood and Tissue kit (Qiagen).

### PCR primers

PCR primers used for this study are in listed in Table S1 in Additional file 1.

### ChromPET library construction

All chromPET libraries were constructed according to the protocol supplied by Illumina with minor modifications. Genomic DNA was extracted with DNAZol reagent and 2 μg of DNA was sheared by a Nebulizer for 5 minutes by compressed air at 32 to 35 psi. After purifying the sample with a QIAquick PCR purification kit, fragmented DNA was run in 2.0% agarose gel, and 0.5-kb fragments were excised from the gel and extracted with a QIAquick Gel Extraction Kit. The ends of DNA fragments were polished by an End-It DNA End Repair Kit and A-tail added to the 3' end by 0.25 units of Taq DNA polymerase. The Y-shaped adapter containing the bar-code was ligated to both ends of DNA fragments by a Quick Ligation Kit and purified again by 2.0% agarose gel electrophoresis and a QIAquick Gel Extraction Kit. Y-shaped adapter ligated DNA was amplified by PCR primer PE1.0 and 2.0 for 15 cycles and the amplified fragment was again purified by 2.0% agarose gel electrophoresis and a QIAquick Gel Extraction Kit. The sequences of adapters and primers are given in Table S1 in Additional file 1.

### RNA bait preparation

We amplified 6.6 kb DNA containing the M-Bcr region from normal lung genomic DNA using PCR primer pair M-BCR-F1 and R1. Amplified DNA (2 μg) was sheared in a Nebulizer for 8 minutes by compressed air at 32 to 35 psi to obtain 0.3-kb fragments, overhanging ends blunted by 2 units of T4 DNA polymerase, the 5' end dephosphorylated by 1 μl of APex Heat-Labile Alkaline Phosphatase, and an A base overhang added to the 3' end by 0.25 units of Taq DNA polymerase. Following each step, the sample was cleaned up by a MinElute Reaction Cleanup Kit. The DNA was cloned into the pCR4-TOPO-TA vector and the resulting construct used to transform *Escherichia coli *competent cells (TOP10). Plasmid DNA was purified from pooled colonies and inserts were amplified by PCR (M13 forward and reverse primer). A 100 μl reaction volume was prepared using 10 ng plasmid DNA, 10 μl 10× Ex Taq Buffer (contains 20 mM MgCl_2_), 2.4 μl 25 mM dNTP solution, 0.6 μl of 100 μM M13 forward and reverse primer sets, 5 U TaKaRa Ex Taq DNA Polymerase and distilled, deionized H_2_O. Repeat-rich DNA (100 ng; human Cot-1 DNA) was also included in the reaction mixture to eliminate repetitive sequences by interfering with extension of the probe across repetitive sequences [19]. The temperature-time cycling profile was as follows: 95°C for 5 minutes followed by 20 cycles of 94°C for 1 minute, 55°C for 20 s and 72°C for 30 s. This was followed by 5 minutes at 72°C and a hold at 4°C until tubes were removed. The DNA was then converted into RNA bait for selection by *in vitro *transcription reaction with Biotin-16-UTP (MAXIscript Kit), following which the DNA template was eliminated by TURBO DNase.

### Anchored ChromPET library preparation

We hybridized 500 ng of biotin-labeled unique single-stranded RNA from the bait to 500 ng of heat-denatured chromPET library in 26 μl of hybridization mixture (5× SSPE, 5× Denhardts', 5 mM EDTA, 0.1% SDS, 20 U SUPERase-In), including 2.5 μg of heat-denatured human Cot-1 DNA and salmon sperm DNA at 65°C for 3 days. RNA-DNA hybrid was captured on Dynabeads M-280 streptavidin that had been washed three times and resuspended in 200 μl of 1 M NaCl, 10 mM Tris-HCl (pH 7.5), 1 mM EDTA and 100 μg/ml salmon sperm DNA. RNA-DNA hybrid capture beads were washed with 0.5 ml of 1× SSC/0.1% SDS once for 15 minutes at 20°C and then with 0.5 ml of 0.1× SSC/0.1% SDS for 15 minutes at 65°C three times. The annealed DNA was eluted by 50 μl of 0.1 M NaOH, neutralized by 70 μl of 1 M tris-HCl (pH 7.5) and converted to double-stranded DNA by paired-end PCR primer PE1.0 and 2.0. DNA fragments were purified by 2.0% agarose gel electrophoresis and high-throughput sequencing was performed according to the manufacturer's protocol (Illumina).

### Bioinformatics pipeline

To identify the sample for each individual chromPET in the multiplexed sequencing runs, we used a 4-bp barcode that was included in the sample-specific Y-primers and was appended to the 5' end of each sequence. Allowing a 1-bp mismatch (only in degenerate positions) the chromPET was assigned to one of the samples or left unassigned. The 38-bp PET reads obtained from the sequencer were mapped to the targeted regions using Novocraft Novoalign program (version 2.05) [20]. We extracted the sequence of the mBCR locus and the sequence of the *ABL1 *gene and indexed them using the Novoindex program (a part of the NovoAlign package). The mapping was done using default mapping parameters (*novoalign *-*r All *-*e 50*). We then used the pipeline as described in [14] to identify chromPETs that have both tags mapping back uniquely to the target regions. The chromPETs were then classified into normal chromPETs (mapping *BCR*-*BCR *and *ABL1*-*ABL1*) and junctional chromPETs (*BCR*-*ABL1 *or *ABL1*-*BCR*). The data discussed in this publication have been deposited in NCBI's Short Read Archive with accession number [SRA023490.1].

### Algorithm for breakpoint prediction

The algorithm for breakpoint detection is based on a voting procedure. We allow each junctional chromPET to vote on the location of the actual breakpoint (Figure S2 in Additional file 1). First, the normal chromPETs for all samples are used to estimate the average and standard deviation of fragment lengths. Using these estimates, each tag of a junctional chromPET votes on the likely location of the breakpoint: vote of 3 to the interval that is the average fragment length downstream of the start of the tag; vote of 2 to the interval one standard deviation down from the end of the 3 zone; and vote of 1 to the interval another standard deviation downstream from the 2 zone. All votes are totaled and plotted over the *BCR *(or *ABL*) locus, and the region with the maximum votes contains the predicted breakpoint. The DNA primers to amplify the junctional fragment (for sequencing across the junction) are designed to encompass this predicted breakpoint-containing region.

### DNA and RNA extraction

DNA and RNA from freshly prepared cell lines, formalin fixed cells, and culture medium were extracted with DNAzol, Trizol, QuickExtract FFPE DNA Extraction Kit, or QuickExtract FFPE RNA Extraction Kit according to the manufacturer's protocol.

## Results

### Effective capture of the target regions and sample multiplexing

**Figure 1:**
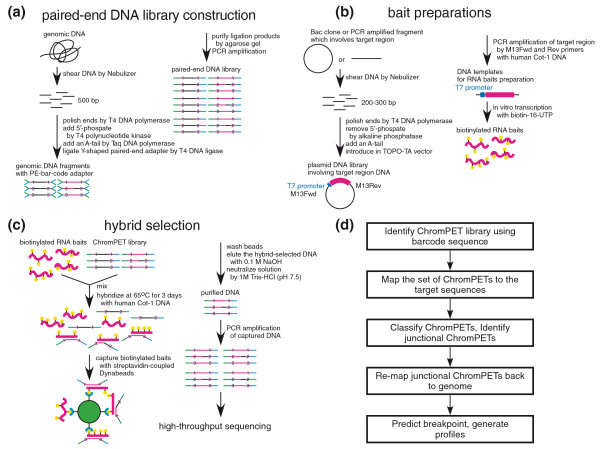
**Outline of Anchored ChromPET method**. Details are in Materials and methods. **(a) **Y-primers containing the sequencing primer and the bar code (1, 2 or 3) ligated to sized genomic fragments. **(b) **RNA bait for anchoring the targeted region prepared by cloning the fragments in a TOPO-TA vector and *in vitro *transcription. **(c) **Y-primed library is selected on the RNA bait, eluted and amplified with paired-end primers to create the bar-coded libraries for paired-end sequencing. **(d) **Bioinformatics pipeline with sequence data.

The chromPET library was constructed according to the manufacturer's protocol with a slight modification. We used Y-shaped adapters that encoded the bar-code sequence immediately after the sequencing primer and before the insert to be sequenced (Figure 1a). Approximately 6.6 kb including the M-bcr region was obtained by PCR from normal lung genomic DNA and converted into a biotinylated RNA bait as described in the methods (Figure 1b). The chromPET library was then hybridized to the RNA bait and purified on streptavidin beads (Figure 1c). We verified that the selection method successfully enriched DNA annealing to the M-bcr region by quantitative real time PCR using primers (M-BCR-F2 and R2) mapping to the 5' region of the M-bcr. The patient samples showed 5,800- to 17,000-fold enrichment of *BCR *DNA by the selection procedure (Figure S1 in Additional file 1).

### Identification of junctional chromPETs

We multiplexed the bar-coded libraries from two leukemia cell lines, K562 and KU812, into one lane and that from three patient samples, PS1, PS2 and PS3, into another lane of the Illumina Genome Analyzer. We performed 38 cycles of paired end sequencing using the protocols provided by the manufacturer.

As shown in Tables 1 and 2, we sequenced 3.2 million 38-bp paired-end reads from the lane with cell lines and approximately 0.5 million 38-bp paired-end reads from the lane with patient samples. The sequenced reads obtained from the Illumina Genome Analyzer were processed through the bioinformatics pipeline as shown in Figure 1d (described in Materials and methods). The resulting chromPETs from the pipeline were classified into two categories: chromPETs that map normally to the *BCR *or the *ABL *region; and junctional chromPETs that map across the junction between *BCR *and *ABL1*.

**Table 1:** Sequencing and mapping numbers for cell lines out of 3,249,760 total reads

	Cell line	
	
	K562	KU812
Barcoded reads	161,365	1,468,876
Mapped		
First tag	24,385	243,684
Second tag	25,310	246,861
Percent mapped		
First tag	15%	17%
Second tag	16%	17%
Mapped uniquely		
First tag	12,800	125,795
Second tag	13,321	122,665
Total anchored chromPETs	2,839	21,798
Junctional chromPETs	131	427
Percent breakpoint	4.6%	2.0%

The number of chromPETs sequenced, mapped, anchored to *BCR *and that were junctional for each cell line.

**Table 2:** Sequencing and mapping numbers for patient samples out of 592,785 total reads

	Cell line		
	
	Patient sample 1	Patient sample 2	Patient sample 3
Barcoded reads	89,316	258,239	37,538
Mapped			
First tag	8,952	30,586	3,782
Second tag	8,861	32,275	3,966
Percent mapped			
First tag	10.0%	11.8%	10.1%
Second tag	9.9%	12.5%	10.6%
Mapped uniquely			
First tag	4,824	16,456	2,186
Second tag	4,828	17,248	2,232
Total anchored chromPETs	994	3,753	403
Junctional chromPETs	23	92	10
Percent breakpoint	2.3%	2.5%	2.5%

Number of chromPETs sequenced, mapped, anchored to BCR and junctional for each sample for patient samples.

Using the criteria on identification of bar-codes described in the Materials and methods, the percentage of chromPETs assigned to each sample was approximately 5% for the K562 cell line and approximately 45% for the KU812 cell line. For the patient samples, the percentages were 15%, 45% and 6% for PS1, PS2 and PS3, respectively. The numbers point to a low efficiency of bar-coding for two of the samples (K562 and PS3), and more study is needed on how to choose uniformly efficient barcodes.

Using default mapping parameters (described in the Materials and methods), we obtained a large but variable number of chromPETs (Tables 1 and 2) anchored in the *BCR *locus (ranging from 21,798 to 403 chromPETs). However, the variable number of sequences mapping to the BCR region allowed us to empirically demonstrate how few sequences were required to use Anchored ChromPET to identify the chromosomal translocation breakpoints. Of the *BCR*-anchored chromPETs, 2 to 4.6% were junctional chromPETs that mapped between the *BCR *and *ABL *loci.

We next devised an algorithm that utilizes the mapping coordinates of each end of a junctional chromPET together with the distribution of sizes of normal chromPETs to predict the most likely position for the breakpoint between the *BCR *and *ABL1 *loci (Figure S2 in Additional file 1; Materials and methods).

Figure S3 in Additional file 1 shows the profile of breakpoint predictions over the M-bcr and *ABL1 *loci for each sample. For the two cell lines and PS1 and PS2, we have well-defined peaks in the breakpoint profile in both the M-bcr and ABL1 *loci*. The locations of these peaks are considered the predicted breakpoints. In contrast, for PS3 the breakpoint predictions are dispersed and do not yield a single peak. The genome coordinates of the predicted breakpoints are shown in Table 3.

**Table 3:** Predicted and actual breakpoints from each sample

	Prediction		Break point	Actual		Difference (bp)	
				
Sample	M-*BCR*	*ABL1*		M-*BCR*	*ABL1*	M-*BCR*	*ABL1*
K562	110,194-110,207	27,762-27,909	*BCR*-*ABL1*	110,191-110,192	27,878-27,879	3	0
KU812	110,241-110,242	63,843-63,853	*BCR*-*ABL1*	110,299-110,300	63,929-63,930	57	76
			*ABL1*-*BCR*	110,096-110,097	63,804-63,805	144	38
Patient 1	109,790-109,830	125,280-125,623	*BCR*-*ABL1*	109,781-109,782	125,326-125,327	8	0
			*ABL1*-*BCR*	109,670-109,671	149,445-149,446	119	^a^23,822
Patient 2	109,702-109,867	102,484-102,653	*BCR*-*ABL1*	109,834-109,835	102,524-102,525	0	0
			*ABL1*-*BCR*	109,869-109,870	102,526-102,527	2	0

Predicted and actual breakpoints for each sample. The absolute difference (in base pairs) between predicted breakpoint site and sequenced breakpoint site is shown in the last two columns. All M-bcr coordinates are relative to chr22:23,522,552 (start position of *BCR *gene). All *ABL1 *coordinates are relative to chr9:133,586,268 (start position of *ABL1 *gene). ^a^We had a secondary peak at this locus in the patient 1 *ABL1 *breakpoint profile (Figure S3D in Additional file 1).

### Prediction and validation of translocation breakpoints in CML cell lines

**Figure 2:**
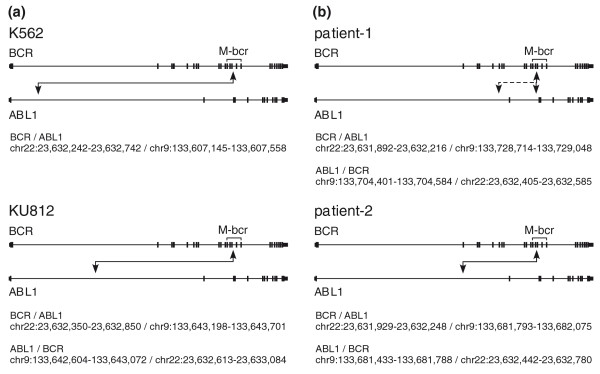
**Predicted junctions between chromosomes 9 and 22**. **(a, b) **Only the *BCR*-*ABL *translocation was detected in K562, but both *BCR*-*ABL1 *and *ABL1*-*BCR *translocations were detected in the KU812 cells and two patient samples. Details of the junctions are in Figure S4 in Additional file 1.

**Figure 3:**
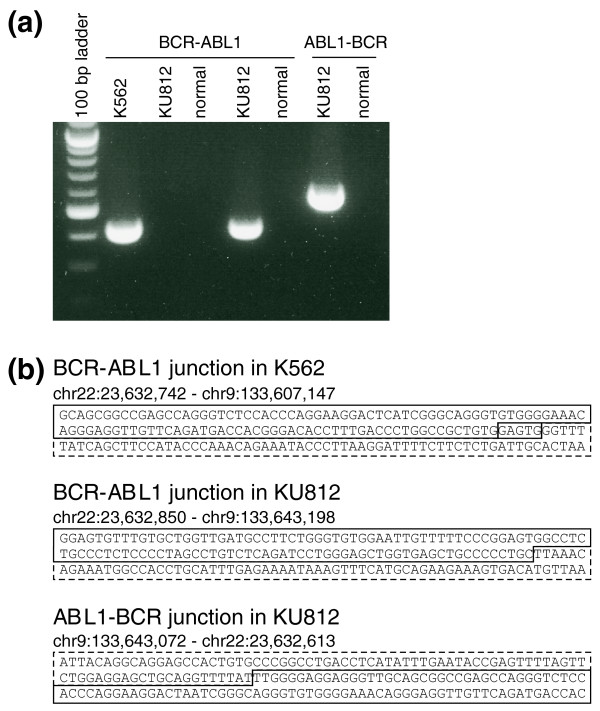
**Validation of predicted breakpoints in cell lines by PCR and Sanger sequencing**. **(a) **Confirmation of chromosome rearrangements by PCR. A primer pair (K562DF1 and R1) yielded a junctional DNA fragment using genomic DNA from K562 (lane 2) but not from normal lung tissues (lane 4). This primer set failed to amplify a DNA fragment using genomic DNA from KU812. PCR primer sets (KU812DF1, R1 and DF2, R2) amplified junctional DNA fragments using genomic DNA prepared from KU812 (lanes 5 and 7) but not from normal lung tissues (lanes 6 and 8). **(b) **Each PCR amplified junctional DNA fragment was cloned into a plasmid vector and Sanger sequencing performed. Solid lines enclose the *BCR *region and broken lines enclose the *ABL1 *region. In K562, a microhomology (GAGTG) exists on the *BCR *and *ABL1 *sides of the breakpoint, so we assume that the ligation point was somewhere in this GAGTG sequence.

The bioinformatics prediction of breakpoints in K562 cells (Table 3 and Figure 2a) agreed well with the breakpoint reported in the literature [21]. To reconfirm this breakpoint, we designed primers flanking these sites and could amplify the junctional fragment from K562 genomic DNA but not from normal lung genomic DNA (Figure 3a). The sequence of the amplified product (Figure 3b) confirmed the reported breakpoint and our bioinformatics prediction.

In a similar fashion we predicted the *BCR*-*ABL1 *junction in KU812 cells (Figure 2a) and confirmed the prediction by amplifying the junctional fragment and sequencing (Figure 3b). Again, our predicted and observed breakpoint agreed with that reported in the literature [21]. We also identified the *ABL1*-*BCR *reciprocal translocation in KU812 cells: sequence tags mapped to chr9:133,642,604-133,643,072 in the *ABL1 *gene were linked to chr22:23,632,613-23,633,084 in the M-bcr (Figure 2a). Again, the predicted *ABL1*-*BCR *junction was confirmed experimentally and found to match exactly with the observed junction (Figure 3b). These data suggest that Anchored ChromPET is capable of identifying gene rearrangements in a targeted region of the genome.

### Prediction and validation of translocation breakpoints in patient samples

We next examined the ability of Anchored ChromPET to identify aberrant translocations in patient samples. To this end, we tested this approach on DNA from blasts in blood samples from Ph+ patients 1 and 2. As a negative control, we also tested this technique in Ph- patient 3. The predicted breakpoints for PS1 and PS2 are reported in Table 3 and Figure 2b.

**Figure 4:**
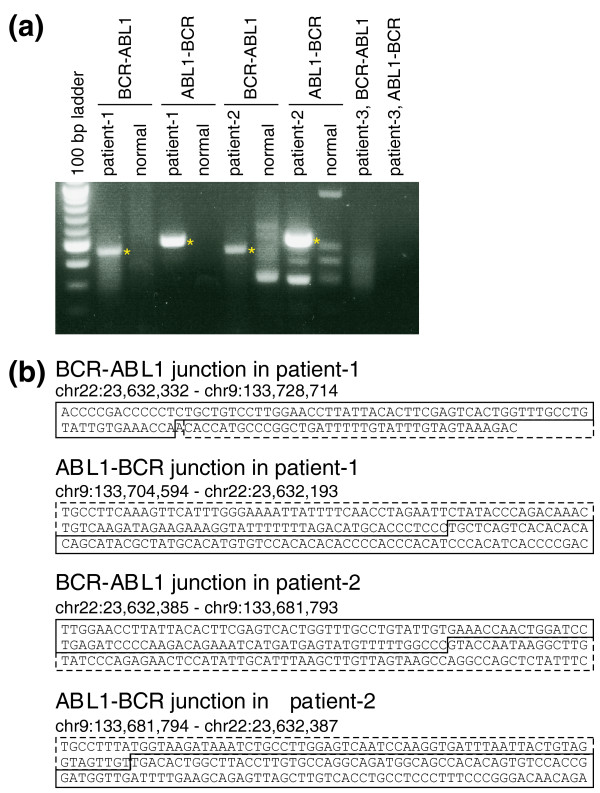
**Validation of predicted breakpoints in patient samples by PCR and Sanger sequencing**. **(a) **Amplified junctional DNA fragments using CML DNA from patients 1, 2, or 3 as template. PCR with primer sets (PhS1F9, R9 and PhS1F2.2, R2.2) successfully amplified a DNA fragment from patient 1 DNA (lanes 2 and 4) but not from patient 3 (lanes 10 and 11). Primer sets (PhS2F1.1, R1.2 and PhS2F2.2, R2.2) gave a product from patient 2 DNA (lanes 6 and 8). The junctional DNA fragment was not detected using genomic DNA from normal lung tissue (lanes 3, 5, 7, and 9). Asterisks indicate unique fragments observed in patients' samples. **(b) **Each PCR-amplified DNA fragment was cloned into a plasmid vector and sequenced. Solid lines enclose the *BCR *region and broken lines enclose the *ABL1 *region.

Based on these results, we designed primer sets, amplified the junctional fragments and confirmed the *BCR*-*ABL1 *and *ABL1*-*BCR *translocations in both these patients. As shown in Figure 4a, predicted junctional fragments were reproducibly amplified from the genomic DNA of patients' blast cells but not from normal lung genomic DNA. Sequencing data for amplified fragments clearly showed the *BCR*-*ABL1 *or *ABL1*-*BCR *junctions in each of these patients (Figure 4b).

A few M-bcr-anchored chromPETs were also linked to the *ABL1 *locus in patient 3, but the predicted breakpoints were dispersed and a unique breakpoint was not predicted using our algorithm. Indeed, PCR with primers spanning the sites that had even the minor peaks (Figure S3C,D in Additional file 1) did not amplify any junctional fragments from the blast cells from patient 3. This suggests that the junctional chromPETs detected were probably due to contamination with PS1 or PS2 DNA during Anchored ChromPET library construction. A retrospective analysis of our protocol indicates that two dispensable steps, both involving gel electrophoresis for size selecting the chromPET library, are the most likely source for this contamination because all three patient libraries were processed simultaneously on the same gel. Of course, we cannot completely exclude the possibility of an atypical *BCR*-*ABL *translocation in patient 3 because the region we have tested is only the 6.6-kb M-bcr. In the future we will expand our anchored area to include the entire *BCR *gene to definitively eliminate the possibility of a *BCR*-*ABL *translocation.

### Comparison of sensitivity: DNA or RNA

**Figure 5:**
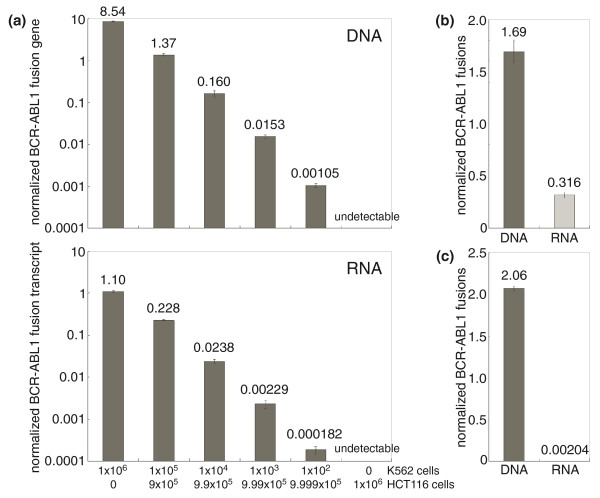
**Sensitivity of detection of DNA junctional fragment**. **(a) **All six samples contained 1 × 10^6 ^cells each, but with a ten-fold serial dilution of K562 cells mixed with an appropriate number of HCT116 cells. The numbers of K562 were 10^6 ^(no dilution), 10^5 ^(1:10), 10^4 ^(1:100), 10^3 ^(1:1,000), 10^2 ^(1:10,000) and 0. Total genomic DNA (100 ng) was used as a template for RT-PCR using PCR primer set K562DF3 and R3. The quantitative PCR signal was normalized to PCR product from the *PCNA *locus. Simultaneously, we isolated total RNA with TRIzol. cDNA reverse transcribed by SuperScript III from 100 ng of total RNA was used as a template for RT-PCR. **(b) **Genomic DNA and RNA were extracted from 10^6 ^formalin fixed KU812 cells. RT-PCR (primer sets KU812DF3, R3 and BCRe13F1, ABL1a2R1) was performed using DNA or cDNA from 10^4 ^cells and normalized to DNA or cDNA from 10^3 ^freshly prepared cells. **(c) **DNA and RNA were prepared from KU812 cell culture medium. DNA or cDNA from 100 μl medium was used for the assay and normalized as above.

Because a clinical sample is not uniformly composed of malignant cells, we next evaluated the sensitivity of detection of the DNA-based biomarkers identified by Anchored ChromPET. A dilution series of K562 cells was created by combining them with HCT116 colon cancer cells without the *BCR*-*ABL1 *translocation. As shown in Figure 5a, we detected the *BCR*-*ABL1 *junctional DNA in 100 ng total DNA even when only 0.01% of the cells carried the *BCR*-*ABL1 *gene and this sensitivity is equivalent to the detection of the fusion transcript in 100 ng RNA by RT-PCR. The sensitivity of the RNA-based RT-PCR methods for detecting *BCR*-*ABL1 *transcripts is similar to that reported in the literature [22].

The most important benefit of Anchored ChromPET is the precise identification of the breakpoints on DNA, which allows for optimal design of PCR primers for a DNA-based biomarker of the translocation junction. It is well known that RNA is less stable than DNA because the 2'-OH group of a ribonucleotide is more reactive than the 2'-H of a deoxyribonucleotide, causing RNA to break more easily, and because RNAses are present on body surfaces and in body fluids. Formalin-fixed, paraffin-embedded (FFPE) tissue is one of the most commonly archived forms for clinical samples. DNA and RNA from FFPE samples are highly fragmented and, in general, the recovery efficiency of DNA is better than that of RNA. Therefore, we evaluated the sensitivity of detection of DNA- or RNA-based junctional biomarkers in samples extracted from formalin-fixed cells. After extraction of DNA or RNA from 10,000 cells, we measured the yield of DNA or RNA junctions by quantitative real-time PCR and normalized the result to the yield from 1,000 fresh cells. As shown in Figure 5b, five-fold more DNA biomarker than RNA biomarker was detected from formalin-fixed cells.

Finally, as cells die they release their DNA and RNA into the body fluids and the ideal biomarker will be stable in serum at body temperature. We therefore measured the amount of DNA or RNA biomarkers that survive in serum-containing cell culture medium at 37°C following the growth of K562 cells (Figure 5c). After filtration of medium to remove cells, we isolated DNA or RNA from 100 μl of medium and measured the amount of junctional biomarker as above. Junctional DNA was detected nearly 10,000 times more efficiently than junctional RNA (Figure 5c), strongly suggesting that the DNA biomarkers identified by Anchored ChromPET will be of great utility for detection of the cancer-derived aberrant DNA in body fluids.

## Discussion

### Advantages of Anchored ChromPET

Anchored ChromPET makes it possible to detect gene rearrangements in a targeted region in a short time and provides a personalized DNA-based biomarker for following a patient's disease. This technique has the advantages of both karyotyping and RT-PCR. Twenty-five to 30 metaphase cells are usually examined during karyotyping so that the sensitivity of detecting a Ph-positive cell is 3 to 4%. Interphase FISH can be applied to nondividing cells isolated from peripheral blood to detect the juxtaposition of *BCR *and *ABL *signals created by a translocation. In this case, about 200 to 500 nuclei are studied, giving a sensitivity of detection of 0.2 to 0.5%. However, the percentage of *BCR*-*ABL1*-positive cells in peripheral blood is lower than that in bone marrow, and the protein digestion step necessary to remove chromatin proteins before FISH affects the signals, making them difficult to interpret. As shown in Table 2, we identified 23 junctional chromPETs from 89,316 reads in PS1, giving an apparent sensitivity of 0.03% for the primary detection of a *BCR*-*ABL *fusion.

We also evaluated the sensitivity of detection of the PCR product spanning the chromosome junction for molecular follow-up of the disease (Figure 5a). The sensitivity of detection of the DNA junction is at least 0.01% and is almost equivalent to that of detecting the RNA fusion. Whereas RNA degradation during sample preparation and silencing of *BCR*-*ABL1 *affect the sensitivity of detection of the fusion RNA [12], the DNA junction is relatively free from these problems.

With G banding, approximately 400 to 800 bands per haploid set can be detected by a trained cytogeneticist. The haploid human genome occupies about 3 × 10^9 ^bp. Thus, the resolution of karyotyping is 5 Mb and the resolution of interphase FISH is 50 to 100 kb. The resolution of RT-PCR for detecting fusion transcripts is not comparable to that obtained here because the chimeric RNA merely indicates the two exons that are fused to each other, with the DNA breakpoints localized anywhere within the adjoining introns. In comparison, we identify the exact DNA junction at the base-pair level by Anchored ChromPET, suggesting that the sequencing-based approach gives the best resolution of the DNA junction.

Anchored ChromPET therefore provides a high-resolution digital karyotype with better sensitivity than comparable methods for detecting the DNA translocation. Note that there is no detectable signal saturation and so the sequencing step can be scaled up by sequencing more DNA to sample even rarer DNA fusion events. About 5 to 10% of CML patients are Ph-negative by karyotyping, but the *BCR*-*ABL1 *transcript is detectable by RT-PCR in half of these cases. In some cases the *ABL1 *gene is inserted in the *BCR *locus and results in the *BCR*-*ABL1 *fusion in a cytogenetically normal chromosome 22 and vice versa [23]. Thus, a significant advantage to DNA sequencing is that we can identify the specific base-pair location of even these chromosome rearrangements. While there is no doubt that CML is caused by the expression of the *BCR*-*ABL1 *fusion transcript, genetic heterogenity of the fusion junction might influence disease progression [13]. Therefore, by giving higher resolution information on the breakpoint compared to an RNA-based method like RT-PCR, Anchored ChromPET may be more useful for future studies correlating the DNA breakpoint with disease progression.

Nondividing cells isolated from peripheral blood, which cannot be used for karyotyping, can be used for Anchored ChromPET. There are reports in the literature of successful isolation of 0.5- to 1-kb DNA fragments from blood smears and formalin fixed paraffin embedded tissue. Therefore, Anchored ChromPET and subsequent PCR detection of junctional DNA can be especially useful for retrospective analysis of patient material for both identification of the translocation and detection of minimal residual disease.

How do we expect this technology to be used in the diagnosis and management of new cases of CML? Most patients present in the chronic phase of CML, characterized by leukocytosis with the presence of precursor cells of the myeloid lineage. There are normally between 4 × 10^9 ^and 1.1 × 10^10 ^white blood cells in a liter of blood, but this number is significantly increased, with up to 10% blast cells and promyelocytes in the blood in chronic phase CML. In acute phase CML more than 70 to 80% of white blood cells in the peripheral blood can be blasts. RT-PCR seems to be the easiest and most sensitive molecular method for detection of the *BCR*-*ABL *transcript in both these situations. Despite this, karyotyping of the bone marrow (or at least interphase FISH of peripheral blood) to detect the fusion at the DNA level is considered the gold standard for diagnosis. We propose Anchored ChromPET as an alternative for detecting the DNA fusion. One milliliter of blood is enough to construct a chromPET library for the identification of the breakpoint, and once a breakpoint is identified PCR will be able to detect gene rearrangements with the same volume of blood. The whole 135 kb of the *BCR *gene can be used as bait, and the resulting 21-fold increase in sequencing is still well within the capability of one-tenth of a lane of a Solexa sequencer, which yields 10 to 20 million reads per lane. An alternative strategy is to use the results of the RT-PCR to define exactly which exon of *BCR *flanks the DNA fusion, and then design a smaller bait that will capture the adjoining intron and junctional DNA fragments to sequence the DNA breakpoint.

A major advantage of Anchored ChromPET is that we do not have to grow the cells in culture and so the method is expected to find wide application in searching for specific translocations for solid cancers where it is difficult to grow all the cancer cells in culture. In addition, since the sensitivity of the method can be increased by sequencing more DNA fragments, we expect it to reliably detect translocations carried by even a small fraction of the cells in a sample. Finally, for translocations (unlike *BCR*-*ABL*) where methods have not been standardized to detect the various alternative fusion transcripts by RT-PCR, Anchored ChromPET can become the method of choice for detecting the DNA fusion that defines the translocation.

Only future experiments will define whether the DNA fusion or the RNA fusion will be the better marker for minimal residual diseases or early recurrence. However, since the detection of the DNA fusion does not need reverse transcription and is not as susceptible to the factors that degrade RNA, we anticipate that the DNA fusion fragment may be a more sensitive biomarker than the RNA fusion fragment. We could easily detect the DNA junctional fragment in filtered cell culture medium, suggesting that DNA derived from dead cells survives in serum at 37°C for an extended period of time. In contrast, it is hard to detect the RNA fusion transcript in the same cell culture medium. This observation suggests that another potential advantage of using the DNA junctional fragment as a biomarker is that it may survive as free nucleic acid in body fluids like blood or even urine. This, again, is something that we are interested in testing in the future.

The decrease in sequencing achieved by anchoring, by sampling only the ends of the fragments and by multiplexing multiple samples in the same lane of a sequencer brings the costs of sequencing down considerably. In our estimate, considering the current state of sequencing capabilities and the small number of sequences necessary to identify the breakpoint, we can reliably multiplex up to ten samples in a single lane of the Illumina sequencer, making the sequencing costs much lower than those for whole genome sequencing for identifying cancer-specific recombination biomarkers.

### Computational prediction of breakpoint

Table 3 shows the coordinates of the predicted breakpoints, the coordinates of the sequenced breakpoints and the difference (in base pairs) between them. For the *BCR *breakpoint in patient 2 cells and *ABL1 *breakpoints in the K562 cell line and patients 1 and 2, the predictions turned out to match exactly to the sequenced breakpoint. Even in other cases, the maximum difference is only 144 bp. In the *BCR*-*ABL1 *fusion in patient 1, a >20-kb deletion in the *ABL1 *locus (Figure S4 in Additional file 1) produced two discrete breakpoint predictions in the *ABL1 *locus (Figure S3 D in Additional file 1) with one corresponding to the *BCR*-*ABL1 *fusion and the other to the *ABL1*-*BCR *fusion.

These results demonstrate that the predictions from our algorithm match reasonably well to the breakpoints verified by experimental methods. Our results also suggest that breakpoints could be predicted using even a small number of junctional chromPETs (K562 and PS1). However, we could not predict a consensus breakpoint from PS3 and could not identify a junctional fragment from this DNA using PCR. So even though junctional chromPETs were assigned to patient 3, these are most likely the result of contamination during chromPET library construction. The fact that the contamination did not lead to a false positive call points to the robustness of the approach.

### Other methods for sequencing the DNA translocation junction

Ligation of a special adapter to the ends of genomic DNA fragments, PCR cycles beginning with an exon of *BCR*, and nested PCR starting with the adapter have been used sequentially to clone and sequence several *BCR*-*ABL *junctions [24]. In another approach, six forward primers were used to cover 3 kb of the M-bcr and 302 reverse primers were used to cover 150 kb of the *ABL *gene so that PCR could be used to identify potential junctions with clever adaptations in order to remove non-specific PCR products [25]. Both these methods, however, can only be used when we know that the breakpoint is close (within a distance suitable for PCR) to a limited part of the *BCR *gene. In comparison, Anchored ChromPET was used in this paper to identify a breakpoint anywhere in the 6 kb M-Bcr region and can be readily scaled up to screen for breakpoints in the entire 135 kb *BCR *gene. The breakpoint on the other side can be anywhere in the *ABL *gene (or for that matter, anywhere else in the genome). Furthermore, as demonstrated here, the method often yields the reciprocal *ABL*-*BCR *junction.

### RNA bait preparation

Well-designed RNA baits useful for the capture of DNA fragments can be commercially synthesized [26]. However, such baits are very expensive, and will be even more expensive if larger parts of the genome need to be anchored. For example, in this paper we used the 6.6 kb region containing M-bcr in chromosome 22q11 as the anchoring DNA, because >90% of CML *BCR *breakpoints are in this region. However, breakpoints in the minor breakpoint cluster region (m-bcr) are seen in ALLs, and are distributed over a 90-kb region in intron 1 of the *BCR *gene. The different method of bait preparation described in this paper is cost-efficient and can be scaled up to cover the whole 135-kb *BCR *gene, which will allow us to identify rare breakpoints in the m-bcr or micro-bcr regions and also to definitively rule out translocations anywhere in the *BCR *gene.

### Translocation junctions

Detection of both reciprocal translocations in KU812 and two patient samples allowed us to analyze what happens to the ends of the chromosomes after the break that initiates the translocation. Some DNA sequence is lost at the *ABL1 *locus in all samples and at the *BCR1 *locus in patient 2, most likely due to exonuclease activity before ligation (Figure S4 in Additional file 1).

In contrast, in KU812 cells and patient 1, some of the DNA at the *BCR *locus seems to be duplicated, so that the *BCR *breakpoint in the *BCR*-*ABL *fusion is downstream of the *BCR *breakpoint in the *ABL*-*BCR *fusion (Figures S3, S4 and S5A in Additional file 1). This kind of duplication is often observed in balanced chromosome rearrangements [27]. DNA mfold [28] predicts that the DNA around the *BCR *breakpoints in KU812 forms a stem-loop structure with a Gibbs free energy (dG) of -88.96 kcal/mol (Figure S5B in Additional file 1). Hairpin- or cruciform-like DNA structures are strongly associated with genomic instability by their interference with DNA replication in both prokaryotes and eukaryotes. It is hypothesized that formation of a stable secondary DNA structure in this region is responsible for the breakpoint in M-bcr [29-31]. If the cruciform breaks at different points on the two strands of *BCR*, the resulting 3' overhang on each strand could be blunted by continued polymerase action (Figure 5c), leading to the duplication of DNA from the *BCR *locus. Such a cruciform structure, however, was not detected around the duplicated region in patient 1, so this may not be the only mechanism for the duplication.

## Conclusions

The detection of the *BCR*-*ABL1 *fusion gene is critical for the diagnosis of chronic myeloid leukemia and for following the progress of patients after therapy. Currently, karyotyping or interphase FISH is considered the gold standard for diagnosis of specific chromosomal translocations. Compared to these methods, paired-end sequencing is highly sensitive for detecting chromosomal translocations, has high resolution, and lends itself to high throughput automation. However, genome-wide sequencing to detect *BCR*-*ABL1 *translocation is too expensive. Therefore, we made genomic DNA libraries with adapters including bar codes and captured the major break cluster region in the *BCR *gene from whole genomic DNA. By paired-end sequencing of such captured libraries we can identify the exact breakpoints in the *BCR *and *ABL1 *genes in two cell lines and two CML patients. We also show that detection of the DNA junctional fragment is comparable in sensitivity to the detection of the RNA fusion transcript by RT-PCR if the RNA is harvested and stored under carefully controlled laboratory conditions. Under non-ideal conditions, such as from formalin-fixed cells or from cell-free nucleic acids in serum, the DNA junctional fragment is more stable and is detected at higher sensitivity. This Anchored ChromPET approach is an efficient method for detecting *BCR*-*ABL1 *and potentially useful for many other chromosomal translocations currently identified by cytogenetics. It has the added advantage of providing a DNA-based biomarker for the translocation that can be used for follow-up of the patient.

## Abbreviations

B-ALL: B-cell acute lymphoblastic leukemia; BP: base pair; CHROMPET: chromosomal paired end tag; CML: chronic myeloid leukemia; FFPE: formalin-fixed: paraffin-embedded; FISH: fluorescent *in situ *hybridization; M-BCR: major breakpoint cluster region; PET: paired-end tag; PH: Philadelphia chromosome; PS: patient sample; RT-PCR: real-time reverse transcription PCR.

## Competing interests

AD in partnership with the University of Virginia has founded a company to commercialize this technology.

## Authors' contributions

All authors contributed to the conception of this project. YS developed Anchored ChromPET library preparations and validated predicted regions by PCR. AM designed a strategy of data analysis. AD devised and supervised the project. All authors contributed to the drafting of the manuscript.

## Supplementary Material

Additional file 1 **Figures S1 to S5 and Table S1**. Figure S1: evaluation of capture efficiencies by quantitative RT-PCR. The fold enrichment of the M-bcr in the libraries prepared from each patient's DNA. Figure S2: a depiction of the algorithm for breakpoint prediction. The schematic illustrates the voting-procedure-based algorithm for breakpoint detection. Figure S3: predicted and actual breakpoints. The UCSC genome browser snapshots from the cell lines and patient samples for the M-bcr locus and *ABL1 *locus. Figure S4: reciprocal translocation breakpoints. The schematic illustrates the duplication or deletion observed in the *BCR *and *ABL1 *breakpoint. Figure S5A: duplicated sequence observed in M-bcr in KU812, showing the 3' end sequence of the breakpoint in the *BCR*-*ABL1 *fusion gene and the 5' end sequence of the breakpoint in the *ABL1*-*BCR *fusion gene. Figure S5B: secondary DNA structure of the sequence that was duplicated in KU812 cells. The MFold-predicted secondary structures of the 638-bp-long sequence, including the duplicated sequence in KU812 cells. Figure S5C: a model for the hairpin-mediated replication fork stalling, asymmetric break on the two strands and sequence duplication. The schematic model of the mechanism of sequence duplication observed in the *BCR*-*ABL1 *breakpoint. Table S1: PCR primers used in this study. Click here for file
