# Supplementary material for: Detection of DNA fusion junctions for BCR-ABL translocations by Anchored ChromPET
Source: Genome Med. 2010 Sep 22;2(9):70. doi: 10.1186/gm191 (PMC3092121; doi:10.1186/gm191)
Supplement: Additional file 1 — Figures S1 to S5 and Table S1. Figure S1: evaluation of capture efficiencies by quantitative RT-PCR. The fold enrichment of the M-bcr in the libraries prepared from each patient's DNA. Figure S2: a depiction of the algorithm for breakpoint prediction. The schematic illustrates the voting-procedure-based algorithm for breakpoint detection. Figure S3: predicted and actual breakpoints. The UCSC genome browser snapshots from the cell lines and patient samples for the M-bcr locus and ABL1 locus. Figure S4: reciprocal translocation breakpoints. The schematic illustrates the duplication or deletion observed in the BCR and ABL1 breakpoint. Figure S5A: duplicated sequence observed in M-bcr in KU812, showing the 3' end sequence of the breakpoint in the BCR-ABL1 fusion gene and the 5' end sequence of the breakpoint in the ABL1-BCR fusion gene. Figure S5B: secondary DNA structure of the sequence that was duplicated in KU812 cells. The MFold-predicted secondary structures of the 638-bp-long sequence, including the duplicated sequence in KU812 cells. Figure S5C: a model for the hairpin-mediated replication fork stalling, asymmetric break on the two strands and sequence duplication. The schematic model of the mechanism of sequence duplication observed in the BCR-ABL1 breakpoint. Table S1: PCR primers used in this study. [file gm191-S1.PDF]

Supplementary data

Figure S1. Evaluation of capture efficiencies by quantitative real time PCR. ChromPET (original) and Anchored ChromPET (captured) libraries prepared from each patient's DNA were used for the evaluation of capture efficiency. PCR primer set (M-BCR-F2 and R2) mapping to the 5' region of M-bcr was used for this experiment. Each signal was normalized to the signal from the PCNA locus (primer set hPCNA-F1 and R1). The target region in patient samples were enriched from 5,800 to 17,000-fold.

Figure S2. A depiction of the algorithm for breakpoint prediction. (A) All tags mapped to the region of interest are identified along with their orientation, (B) Each tag contributes a decreasing vote to basepairs downstream of the tag, the vote decrease the farther one is from the starting position of the tag, (C) All votes are aggregated over the region and the region with the maximum votes is called the predicted breakpoint.

Figure S3. Predicted and actual breakpoints. UCSC genome browser snapshots from the Cell Lines for (A) the M-bcr locus and (B) ABL1 locus, indicating the predicted breakpoint location and the position of the sequenced breakpoints. (C) M-bcr locus and (D) ABL1 locus showing similar information for the three patient samples. The absence of a single dominant predicted breakpoint

in patient sample 3 alerted us to the possibility of a contamination leading to the contaminating junctional chromPETs.

Figure S4. Reciprocal translocation breakpoints. chr22:23,632,613-23,632,850 region of BCR gene in KU812, chr22:23,632,193-23,632,332 region of BCR gene in patient-1, and chr9:133,681,793-133,681,794 region in ABL1 gene in patient-2 were duplicated. 1 bp deletion (chr22:23,632,386) was found in BCR breakpoint in patient-2. Light gray thick lines: part of BCR-ABL1 fusion gene. Black thick line: part of ABL1-BCR fusion gene. Dark gray thick line: part that is represented in both fusions and so indicates a duplication. Thin line: deletion. Lines with arrows: junction for BCR-ABL1 (downward arrow) or ABL1-BCR (upward arrow) fusion genes.

Figure S5A. Duplicated sequence observed in M-bcr in KU812. 3' end of upper sequence indicates the breakpoint in BCR-ABL1 fusion gene and 5' end of lower sequence shows the breakpoint in ABL1-BCR fusion gene. 238 bp area (chr22:23,632,613-23,632,850) contained in solid line was duplicated in KU812 and present in both fusion genes.

Figure S5B. Secondary DNA structure of the sequence that was duplicated in the BCR locus and present in both BCR-ABL and ABL-BCR fusion in KU812 cells. Sequence was computed by DNA mfold. The Gibbs free energy (dG) of this region was -88.96 kcal/mol.

Figure S5C. A model for the hairpin-mediated replication fork stalling, asymmetric break on the two strands and sequence duplication. Light gray thick lines: regions forming cruciform structures. Gray circles: DNA replication machinery. Arrows: leading or lagging strand. Arrowheads: asymmetric breaks in the two strands.

Table S1. PCR primers used in this study.

Figure S1

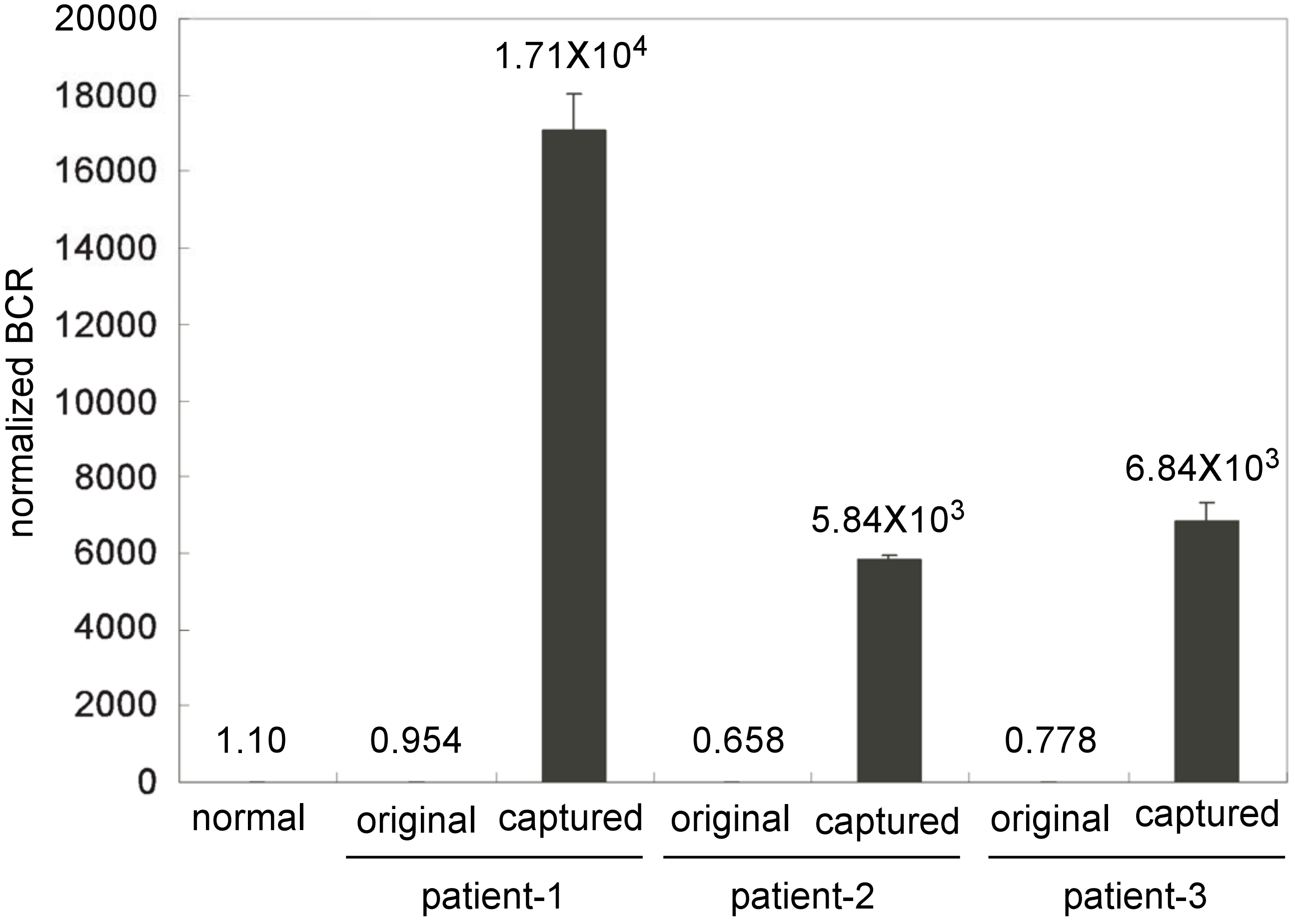
normalized BCR
20000
18000
16000
14000
12000
10000
8000
6000
4000
2000
0
1.71X10^4
5.84X10^3
6.84X10^3
1.10
0.954
0.658
0.778
normal
original
captured
original
captured
original
captured
patient-1
patient-2
patient-3

Figure S2

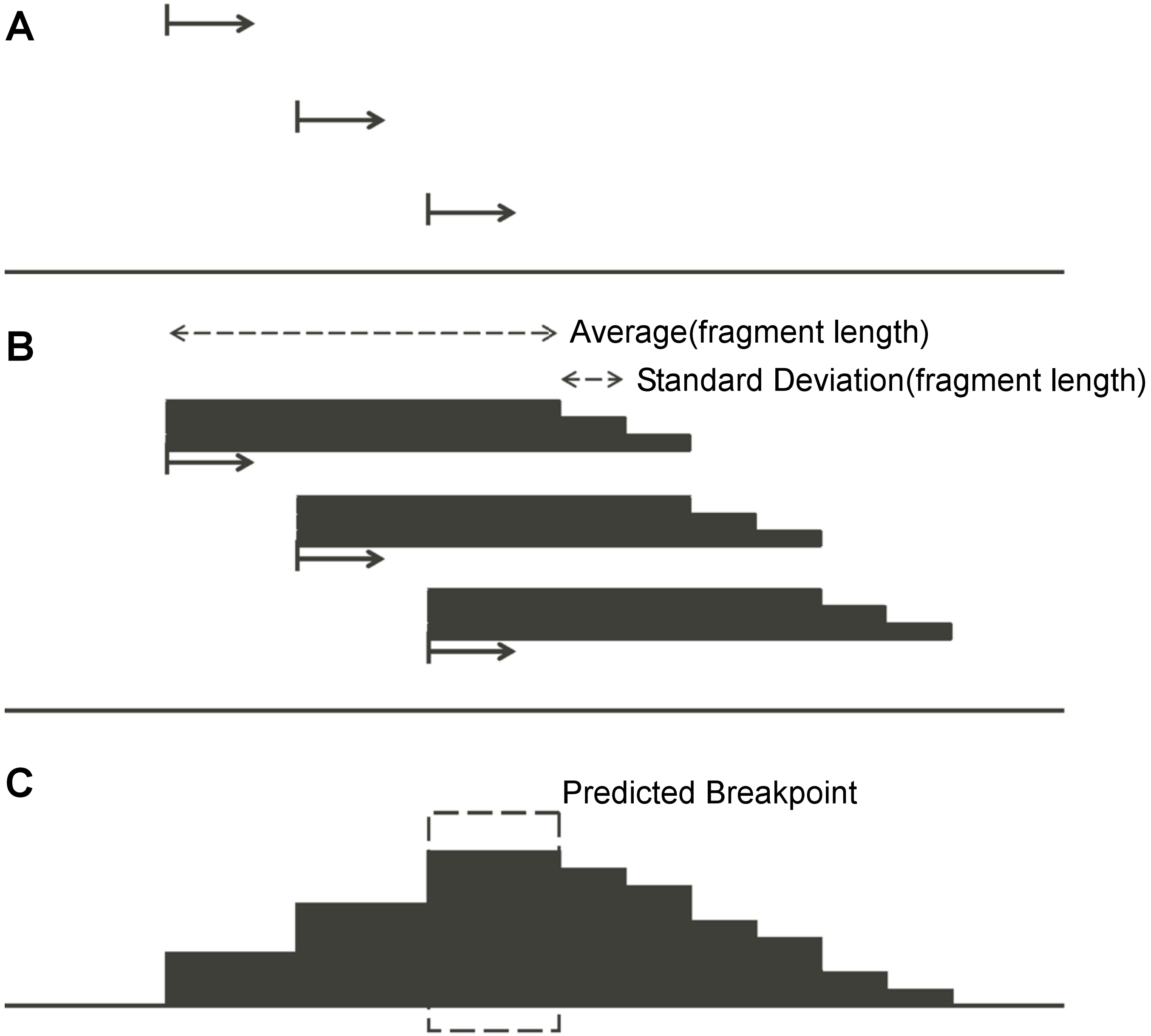
A
B
Average(fragment length)
Standard Deviation(fragment length)
C
Predicted Breakpoint

Figure S3

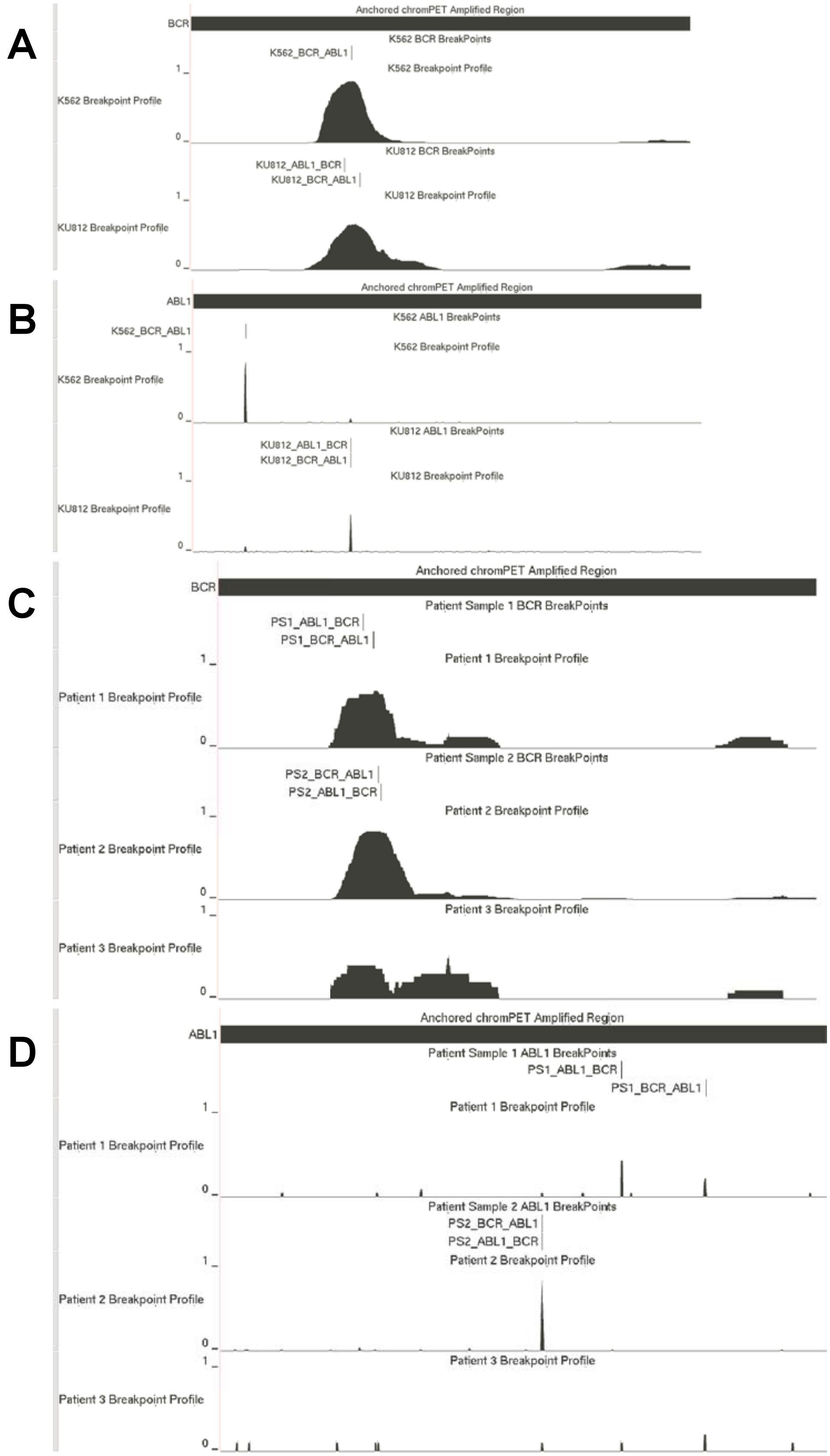

A
Anchored chromPET Amplified Region
BCR
K562 BCR BreakPoints
K562_BCR_ABL1
K562 Breakpoint Profile
K562 Breakpoint Profile
KU812 BCR BreakPoints
KU812_ABL1_BCR
KU812_BCR_ABL1
KU812 Breakpoint Profile
KU812 Breakpoint Profile
B
Anchored chromPET Amplified Region
ABL1
K562 ABL1 BreakPoints
K562_BCR_ABL1
K562 Breakpoint Profile
K562 Breakpoint Profile
KU812 ABL1 BreakPoints
KU812_ABL1_BCR
KU812_BCR_ABL1
KU812 Breakpoint Profile
KU812 Breakpoint Profile
C
Anchored chromPET Amplified Region
BCR
Patient Sample 1 BCR BreakPoints
PS1_ABL1_BCR
PS1_BCR_ABL1
Patient 1 Breakpoint Profile
Patient 1 Breakpoint Profile
Patient Sample 2 BCR BreakPoints
PS2_BCR_ABL1
PS2_ABL1_BCR
Patient 2 Breakpoint Profile
Patient 2 Breakpoint Profile
Patient 3 Breakpoint Profile
Patient 3 Breakpoint Profile
D
Anchored chromPET Amplified Region
ABL1
Patient Sample 1 ABL1 BreakPoints
PS1_ABL1_BCR
PS1_BCR_ABL1
Patient 1 Breakpoint Profile
Patient 1 Breakpoint Profile
Patient Sample 2 ABL1 BreakPoints
PS2_BCR_ABL1
PS2_ABL1_BCR
Patient 2 Breakpoint Profile
Patient 2 Breakpoint Profile
Patient 3 Breakpoint Profile
Patient 3 Breakpoint Profile

Figure S4

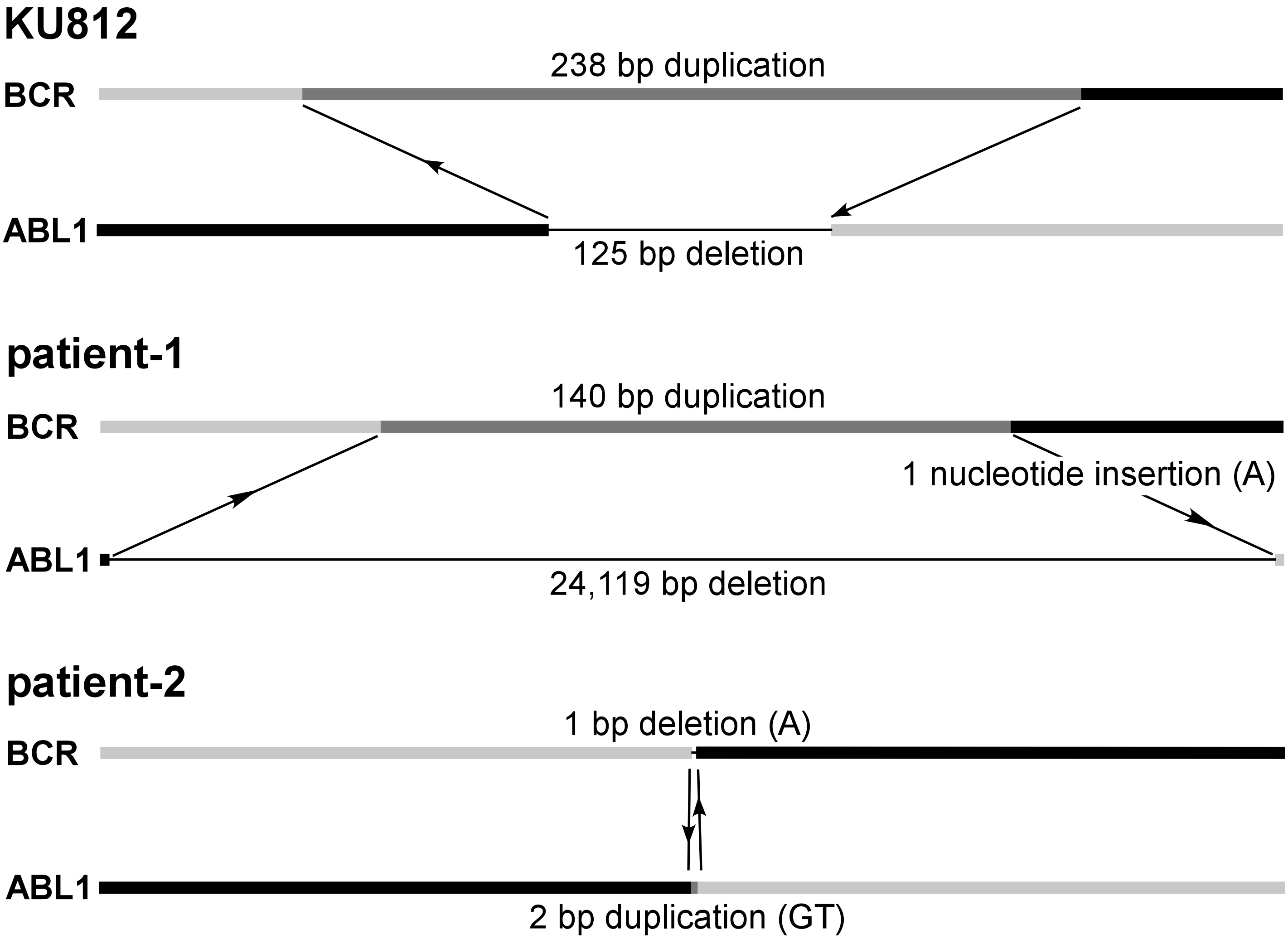
KU812
238 bp duplication
BCR
ABL1
125 bp deletion
patient-1
140 bp duplication
BCR
1 nucleotide insertion (A)
ABL1
24,119 bp deletion
patient-2
1 bp deletion (A)
BCR
ABL1
2 bp duplication (GT)

Figure S5A

3‘ end of BCR in BCR-ABL1 fusion:

chr22:23,632,350-23,632,850

```
CCAAGACAGAAATCATGATGAGTATGTTTTTGGCCCATGACACTGGCTTACCTTGTGCCA
GGCAGATGGCAGCCACACAGTGTCCACCGGATGGTTGATTTTGAAGCAGAGTTAGCTTGT
CACCTGCCTCCCTTTCCCGGGACAACAGAAGCTGACCTCTTTGATCTCTTGCGCAGATGA
TGAGTCTCCGGGGCTCTATGGGTTTCTGAATGTCATCGTCCACTCAGCCACTGGATTTAA
GCAGAGTTCAAGTAAGTACTGGTTTGGGGAGGAGGGTTGCAGCGGCCGAGCCAGGGTCTC
CACCCAGGAAGGACTCATCGGGCAGGGTGTGGGGAAACAGGGAGGTTGTTCAGATGACCA
CGGGACACCTTTGACCCTGGCCGCTGTGGAGTGTTTGTGCTGGTTGATGCCTTCTGGGTG
TGGAATTGTTTTTCCCGGAGTGGCCTCTGCCCTCTCCCCTAGCCTGTCTCAGATCCTGGG
AGCTGGTGAGCTGCCCCCTGC
```

5‘ end of BCR in ABL1-BCR fusion:

chr22:23,632,613-23,633,084

```
TTGGGGAGGAGGGTTGCAGCGGCCGAGCCAGGGTCTCCACCCAGGAAGGACTCATCGGGC
AGGGTGTGGGGAAACAGGGAGGTTGTTCAGATGACCACGGGACACCTTTGACCCTGGCCG
CTGTGGAGTGTTTGTGCTGGTTGATGCCTTCTGGGTGTGGAATTGTTTTTCCCGGAGTGG
CCTCTGCCCTCTCCCCTAGCCTGTCTCAGATCCTGGGAGCTGGTGAGCTGCCCCCTGCAG
GTGGATCGAGTAATTGCAGGGGTTTGGCAAGGACTTTGACAGACATCCCCAGGGGTGCCC
GGGAGTGTGGGGTCCAAGCCAGGAGGGCTGTCAGCAGTGCACCTTCACCCCACAGCAGAG
CAGATTTGGCTGCTCTGTCGAGCTGGATGGATACTACTTTTTTTTTCCTTTCCCTCTAAG
TGGGGGTCTCCCCCAGCTACTGGAGCTGTCAGAACAGTGAAGGCTGGTAACA
```

Figure S5B

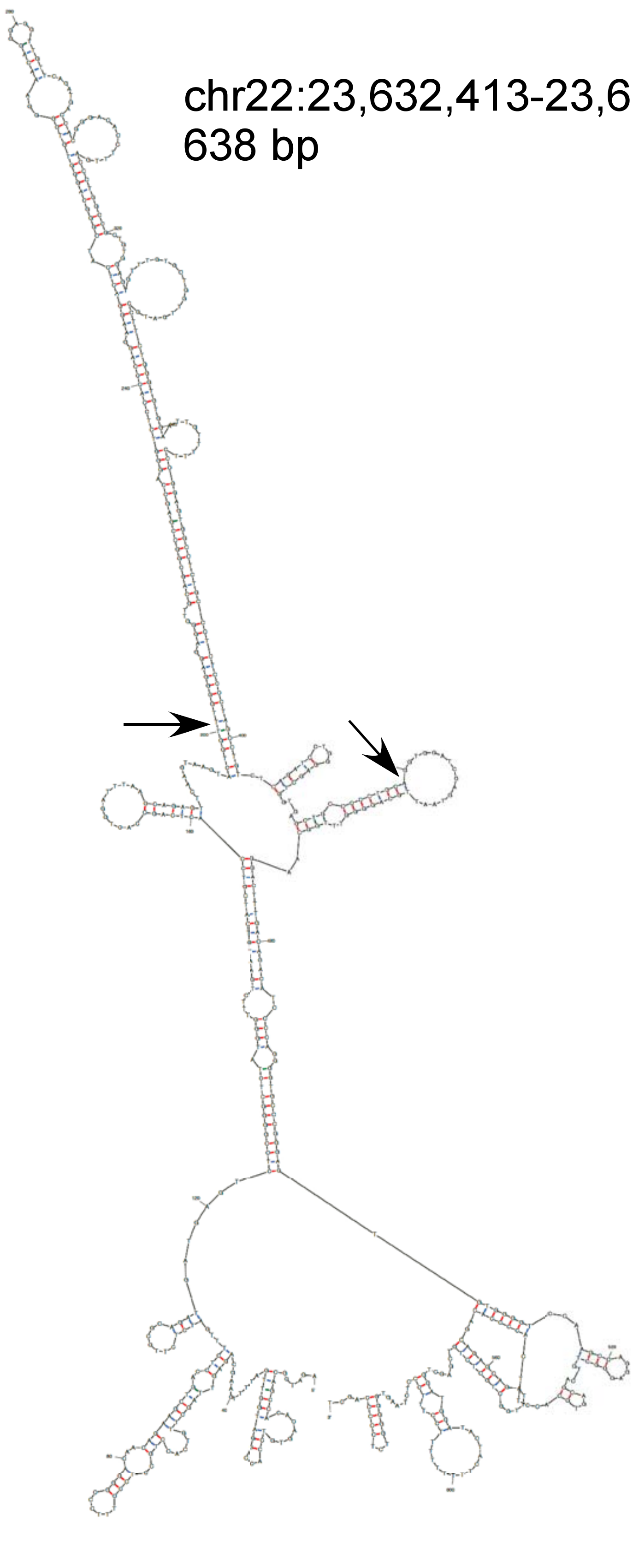

Figure S5C

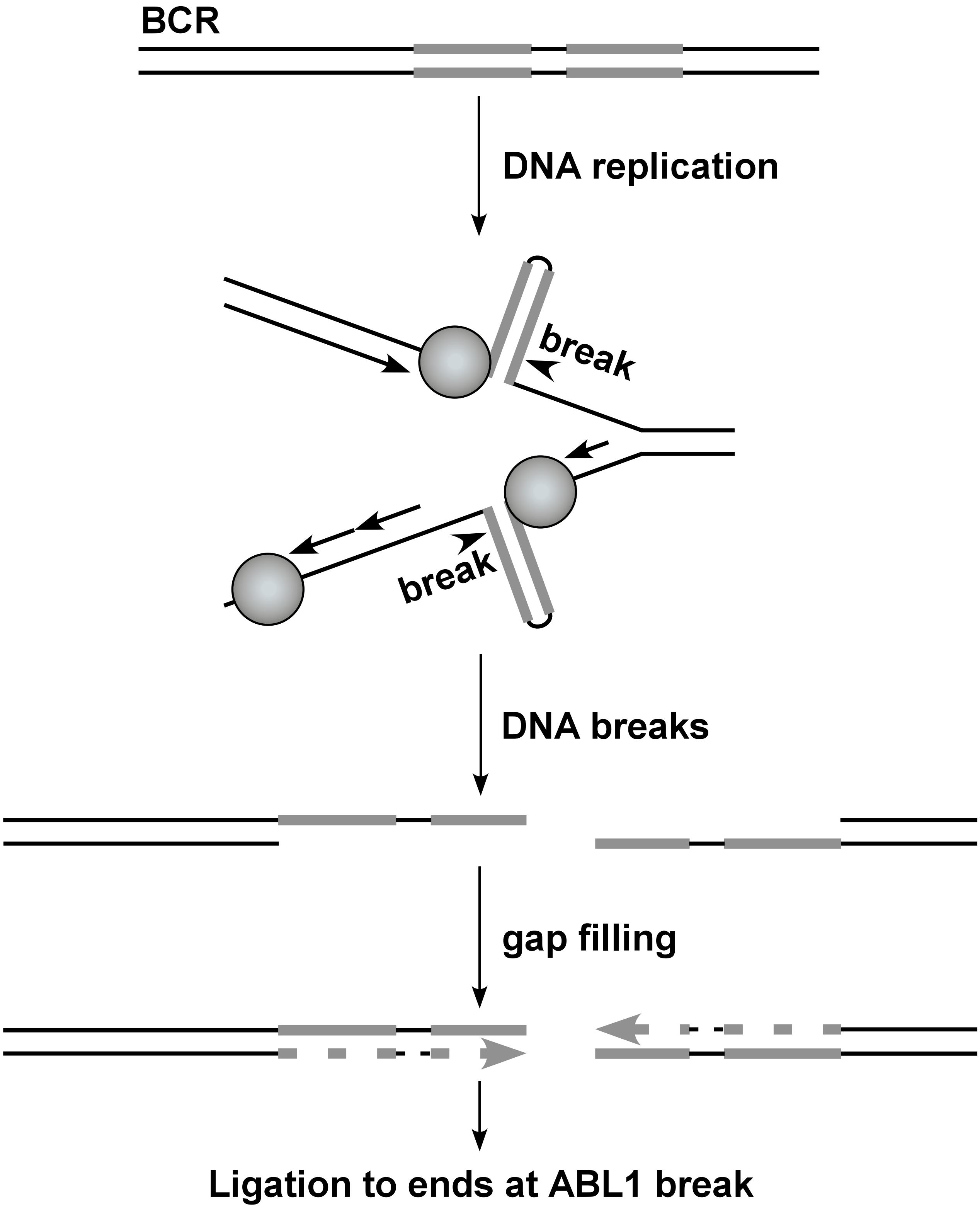
BCR
DNA replication
break
break
DNA breaks
gap filling
Ligation to ends at ABL1 break

Table S1 PCR primers and adapters used in this study

primer name	sequence
M13 Forward<br>M13 Reverse	GTAAAACGACGGCCAG<br>CAGGAAACAGCTATGAC
M-BCR-F1<br>M-BCR-R1	CAGGCCCTTTCCAGATTCCACACCT<br>CCCCAAGGGAGAAGGGAAGTCCAGT
M-BCR-F2<br>M-BCR-R2	CCCAGGGTTTCCTGTCATAACATAG<br>GTGAGGAAAAGGGGCTTATTTCTG
K562DF1<br>K562DR1	TCCACTCAGCCACTGGATTTAAGCA<br>GGTGAATTGGAAAGAAGCAGCAGGT
K562DF3<br>K562DR3	AAACAGGGAGGTTGTTCAGATGAC<br>AAGGGTATTTCTGTTTGGGTATGGA
KU812DF1<br>KU812DR1	GAATGTCATCGTCCACTCAGCCACT<br>TTGTTGTGCAGAATTCCCACCAGTC
KU812DF2<br>KU812DR2	TGGCCTCAGAATGCCATGTTACAGT<br>AACAATTCCACACCCAGAAGGCATC
KU812DF3<br>KU812DR3	CCTTCTGGGTGTGGAATTGT<br>TCACTTTCTTCTGCATGAAACTTTA
PhS1F9<br>PhS1R9	ATGGGACTAGTGGACTTTGG<br>GTCTTTACTACAAATACAAAAATCAGC
PhS1F2.2<br>PhS1R2.2	TGCCTTCAAAGTTCATTTGGGAAAA<br>AGTGGCTGAGTGGACGATGACATTC
PhS2F1.2<br>PhS2R1.2	CAAGCTGTTTTGCATTCACTGTTGC<br>GTCTTGAACTTCTGGGGCTCAAGTG
PhS2F2.2<br>PhS2R2.2	TTCAACCCACAAGGAGCTCACAGTC<br>AAGGCATCAACCAGCACAAACACTC
BCRe13F1<br>ABL1a2R1	AGCATTCCGCTGACCATCAATAAGG<br>GGCCACAAAATCATACAGTGCAACG
hPCNA-F1<br>hPCNA-R1	GTGGTCGTTGTCTTTCTAGGTCTCA<br>GGAAGGAGGAAAGTCTAGCTGGTTT
PCR primer PE 1.0<br>PCR primer PE 2.0	AATGATACGGCGACCACCGAGATCTACACTCTTTCCCTACACGACGCTCTTCCGATCT<br>CAAGCAGAAGACGGCATACGAGATCGGTCTCGGCATTCCTGCTGAACCGCTCTTCCGATCT
PEadapters2	5'p-TCGAGATCGGAAGAGCGGTTCAGCAGGAATGCCGAG 3'<br>3' TAGCTCTAGCCTTCTCGCAGCACATCCCTTTCTCACA 5'
PEadapters3	5'p-GCTAGATCGGAAGAGCGGTTCAGCAGGAATGCCGAG 3'<br>3' TCGATCTAGCCTTCTCGCAGCACATCCCTTTCTCACA 5'
PEadapters4	5'p-ACCAGATCGGAAGAGCGGTTCAGCAGGAATGCCGAG 3'<br>3' TTGGTCTAGCCTTCTCGCAGCACATCCCTTTCTCACA 5'
